# Sterically Stabilized Diblock Copolymer Nanoparticles Enable Convenient Preparation of Suspension Concentrates Comprising Various Agrochemical Actives

**DOI:** 10.1021/acs.langmuir.1c03275

**Published:** 2022-02-22

**Authors:** Derek H. H. Chan, Oliver J. Deane, Emily L. Kynaston, Christopher Lindsay, Philip Taylor, Steven P. Armes

**Affiliations:** † Dainton Building, Chemistry Department, University of Sheffield, Brook Hill, Sheffield, South Yorkshire S3 7HF, U.K.; ‡ Syngenta, Jealott’s Hill International Research Centre, Bracknell, Berkshire RG42 6EY, U.K.

## Abstract

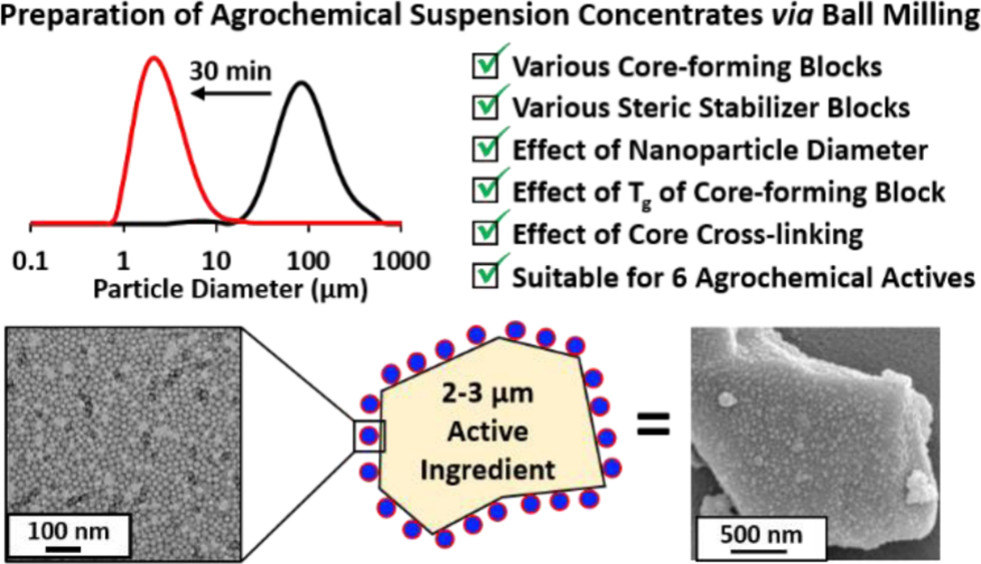

It is well known that sterically stabilized diblock copolymer nanoparticles can be readily prepared using polymerization-induced self-assembly. Recently, we reported that such nanoparticles can be employed as a dispersant to prepare micron-sized particles of a widely used fungicide (azoxystrobin) via ball milling. In the present study, we examine the effect of varying the nature of the steric stabilizer block, the mean nanoparticle diameter, and the glass transition temperature (*T*_g_) of the core-forming block on the particle size and colloidal stability of such azoxystrobin microparticles. In addition, the effect of crosslinking the nanoparticle cores is also investigated. Laser diffraction studies indicated the formation of azoxystrobin microparticles of approximately 2 μm diameter after milling for between 15 and 30 min at 6000 rpm. Diblock copolymer nanoparticles comprising a non-ionic steric stabilizer, rather than a cationic or anionic steric stabilizer, were determined to be more effective dispersants. Furthermore, nanoparticles of up to 51 nm diameter enabled efficient milling and ensured overall suspension concentrate stability. Moreover, crosslinking the nanoparticle cores and adjusting the *T*_g_ of the core-forming block had little effect on the milling of azoxystrobin. Finally, we show that this versatile approach is also applicable to five other organic crystalline agrochemicals, namely pinoxaden, cyproconazole, difenoconazole, isopyrazam and tebuconazole. TEM studies confirmed the adsorption of sterically stabilized nanoparticles at the surface of such agrochemical microparticles. The nanoparticles are characterized using TEM, DLS, aqueous electrophoresis and ^1^H NMR spectroscopy, while the final aqueous’ suspension concentrates comprising microparticles of the above six agrochemical actives are characterized using optical microscopy, laser diffraction and electron microscopy.

## Introduction

Many types of agrochemicals, for example, fungicides, herbicides or insecticides, are organic crystalline compounds with relatively low solubility in aqueous solution.^1^ Traditionally, ball milling has been employed to produce crystalline microparticles of such active ingredients (AIs) in the form of aqueous suspension concentrates (SCs).^2^ This processing technique has been used for several decades to ensure the efficient delivery of AIs to various crops—indeed, this is probably the most widely used formulation within the agrochemical industry. The initial coarse particulates are subjected to wet milling in the presence of a suitable surfactant and/or water-soluble polymer, which acts as a dispersant. Such copolymers enhance the milling efficiency and are essential for conferring steric stabilization to prevent agglomeration or crystal growth.^3^ The final mean microparticle diameter is usually targeted to be ≈2 μm.^4^

Within the last two decades, polymerization-induced self-assembly (PISA) has become widely recognized as a versatile platform technology for the efficient synthesis of many types of block copolymer nano-objects in the form of concentrated dispersions in various solvents.^5−17^ Depending on their copolymer morphology, various applications have been explored for such nano-objects. For example, spherical nanoparticles have been evaluated as emulsifiers for Pickering nanoemulsions^18−20^ or as lubricants for ultralow viscosity automotive engine oils,^21^ worms have been examined as thickeners for silicone oil^22^ or aqueous media^23^ and also as biocompatible gels for stem cell storage^24^ or 3D cell culture,^19^ while vesicles have been used to encapsulate either enzymes or nanoparticles.^25,26^ One of the most commonly reported PISA formulations is RAFT aqueous emulsion polymerization, which is applicable to various water-immiscible commodity vinyl monomers such as styrene, *n*-butyl acrylate, vinyl acetate, or methyl methacrylate.^27−37^ Of particular importance for the present study, such formulations enable the convenient synthesis of sterically stabilized diblock copolymer spheres of tunable size with mean diameters ranging from 20 to 200 nm depending on the degree of polymerization (DP) that is targeted for the hydrophobic core-forming block.^18,38^

Recently, we reported that hydroxyl-functional diblock copolymer nanoparticles can serve as an effective dispersant to prepare SCs comprising micrometer-sized particles of a widely used fungicide (azoxystrobin) via ball milling.^39^ In principle, such sterically stabilized nanoparticles should act as a milling aid while simultaneously conferring long-term steric stabilization. Moreover, hydroxyl-functional nanoparticles are likely to produce SCs exhibiting superior temperature stability and greater salt tolerance compared to copolymer surfactants based on poly(ethylene glycol). In our prior study, poly(glycerol monomethacrylate) (PGMA) was employed as a non-ionic steric stabilizer block, while the hydrophobic core-forming block was either poly(methyl methacrylate) (PMMA) or poly(2,2,2-trifluoroethyl methacrylate) (PTFEMA). In both cases, it was shown that the nanoparticles survived the ball milling process and absorbed intact at the surface of the azoxystrobin microparticles. For the PGMA-PMMA nanoparticles, supernatant assays based on solution densitometry measurements indicated a low-affinity Langmuir adsorption isotherm (with an adsorbed amount, Γ, of approximately 5.5 mg m^–2^), while XPS analysis suggested a fractional surface coverage of 0.24. Nevertheless, aqueous electrophoresis studies confirmed that this relatively low coverage was sufficient to significantly reduce the anionic character exhibited by the nanoparticle-coated azoxystrobin microparticles relative to that of azoxystrobin alone.

**Figure 1:**
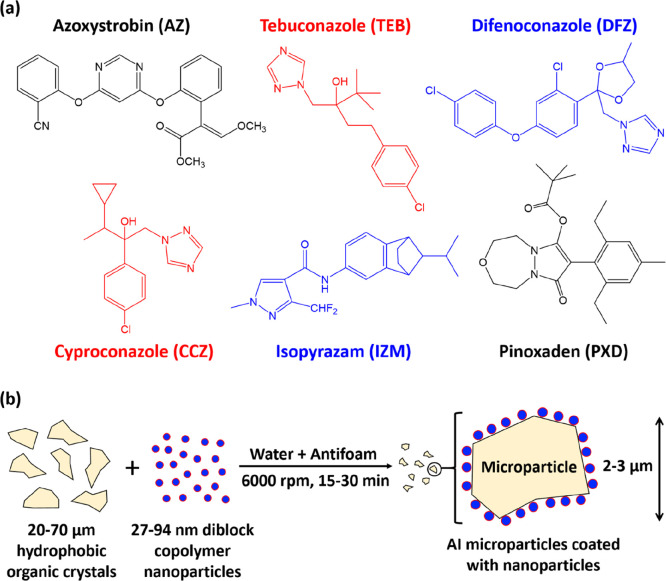
(a) Chemical structures of the six agrochemical active compounds examined in this study: AZ, TEB, DFZ, CCZ, IZM, and PXD. The latter compound is an herbicide, while the other five compounds are fungicides. (b) Schematic cartoon of the preparation of an SC comprising an agrochemical AI in the form of microparticles using sterically stabilized diblock copolymer nanoparticles as the sole dispersant. An IKA Ultra-Turrax Tube Drive containing 1.0 mm ceramic beads was used to mill the initial coarse AI crystals. [N.B. components are not drawn to scale.]

In the present study, we examine how varying the nature of the steric stabilizer block, adjusting the mean nanoparticle diameter, and crosslinking the nanoparticle cores affect the size of the azoxystrobin microparticles. In addition, we briefly explore whether varying the glass transition temperature (*T*_g_) of the core-forming block affects their formation and colloidal stability. Moreover, we demonstrate that this versatile approach is also applicable to a further five widely used agrochemicals, namely pinoxaden (PXD), cyproconazole (CCZ), difenoconazole (DFZ), isopyrazam (IZM), and tebuconazole (TEB), see Figure 1a. The physicochemical properties for all six agrochemical actives used in this study are summarized in Table S1. The various types of diblock copolymer nanoparticles are characterized using TEM, DLS, aqueous electrophoresis and ^1^H NMR spectroscopy, while the aqueous SCs comprising microparticles of the above six agrochemical actives are characterized using optical microscopy, laser diffraction and TEM. Full experimental details for all the PISA formulations and analytical techniques employed in this study can be found in the Supporting Information.

## Results and Discussion

Initially, we sought to extend our prior study by examining how adjusting various synthesis parameters affected the preparation of aqueous SCs comprising azoxystrobin, a widely used fungicide.^39^ Preparation of SC formulations involves milling relatively coarse (20–76 μm diameter) hydrophobic organic crystals in the presence of a suitable polymeric dispersant (Figure 1b). It is perhaps worth mentioning that a control experiment performed in the absence of any dispersant resulted in poor milling efficiency (ca. 10 μm diameter) and excess foam in the case of azoxystrobin. This confirmed that a suitable polymeric dispersant was required during ball milling. In the present study, an IKA Ultra-Turrax Tube Drive was used for milling rather than a planetary ball mill. This approach enabled the convenient preparation of SCs on a relatively small scale. Following our recent publication, a series of sterically stabilized nanoparticles were employed as a dispersant, rather than conventional commercially available water-soluble polymers such as Morwet D-425.^39^

### Effect of Varying the Chemical Nature of the Steric Stabilizer Block

**Figure 2:**
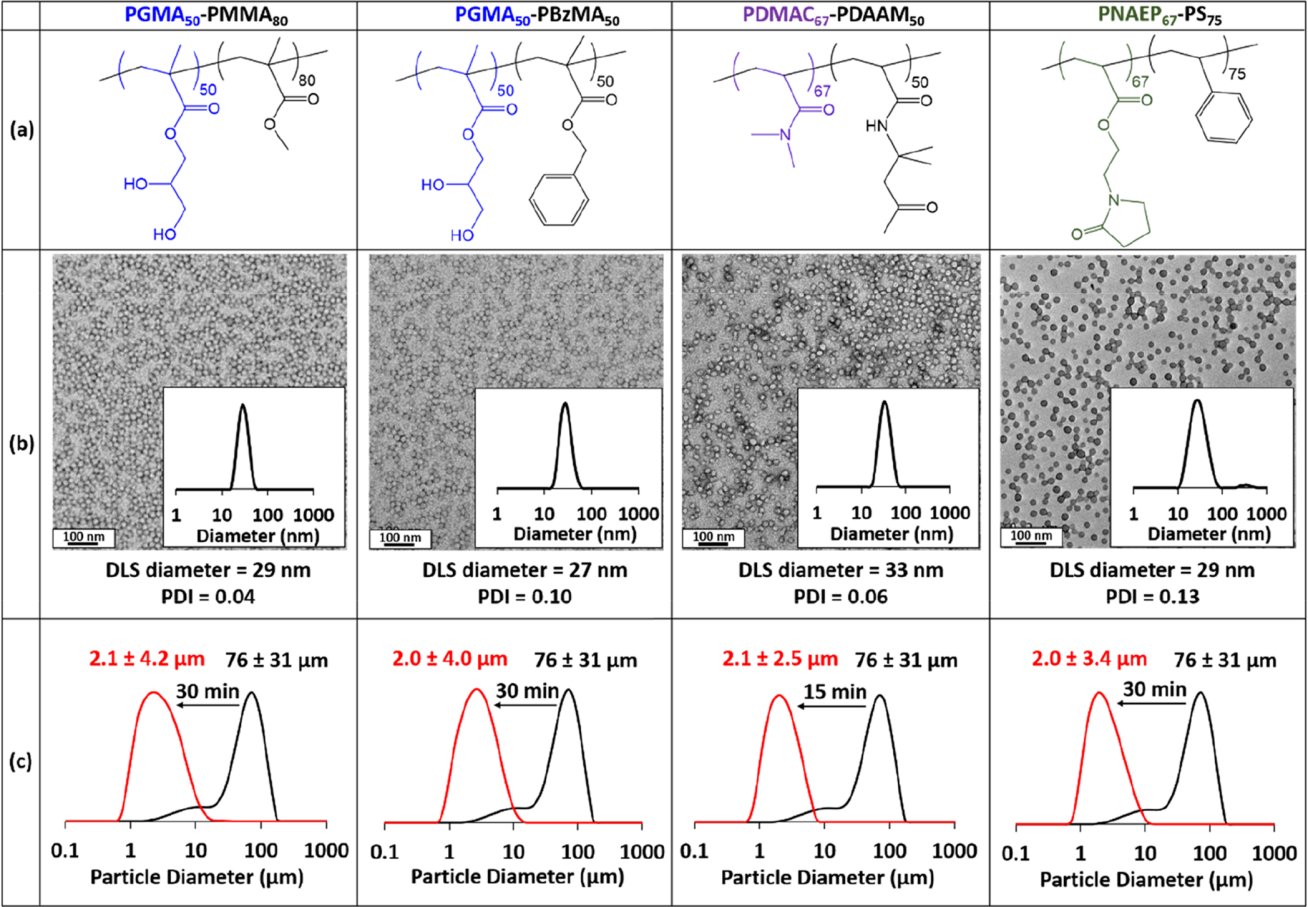
(a) Chemical structures of four of the non-ionic sterically stabilized diblock copolymer nanoparticles used in this study (i.e., PGMA_50_-PMMA_80_,^40^ PGMA_50_-PBzMA_50_,^18^ PDMAC_67_-PDAAM_50,_^41^ and PNAEP_67_-PS_75_^33^). (b) TEM images and DLS intensity-average particle size distributions (see insets) recorded for each type of nanoparticle. (c) Laser diffraction particle size distribution curves (and corresponding volume-average diameters) recorded for unmilled coarse azoxystrobin crystals (black trace) and milled azoxystrobin microparticles (red traces) prepared when using such nanoparticles as the sole dispersant.

Four different types of sterically stabilized nanoparticles were prepared via RAFT polymerization using aqueous PISA formulations described in the literature.^18,33,40,41^ Three non-ionic steric stabilizer blocks were employed, and the relevant chemical structures for the resulting amphiphilic diblock copolymers (PGMA_50_-PMMA_80_,^40^ PGMA_50_-PBzMA_50_,^18^ PDMAC_67_-PDAAM_50_,^41^ and PNAEP_67_-PS_75_^33^) are shown in Figure 2a. TEM studies confirmed that a well-defined spherical morphology was obtained in each case, and DLS measurements indicated that these diblock copolymer nanoparticles had comparable hydrodynamic *z*-average diameters (27–33 nm) and relatively low polydispersities (0.04 < PDI < 0.13), see Figure 2b.

Coarse, polydisperse azoxystrobin crystals of approximately 76 μm diameter were milled in the presence of a 2.5% w/w aqueous dispersion of nanoparticles until a volume-average particle diameter of approximately 2 μm was achieved as judged by laser diffraction studies (Figure 2c). Very recently, we reported successful planetary ball milling of azoxystrobin in the presence of PGMA_50_-PMMA_80_ nanoparticles within 10 min.^39^ In the same study, we found that changing the hydrophobic core-forming block from PMMA to PTFEMA had no discernible effect on either the milling efficiency or the final size of the azoxystrobin microparticles. Similar results were obtained herein when replacing the PMMA core-forming block with PBzMA. More specifically, a final azoxystrobin microparticle diameter of approximately 2 μm was produced within a milling time of 30 min when using PGMA_50_-PBzMA_50_ nanoparticles as a dispersant.

The effect of varying the nature of the non-ionic steric stabilizer was examined by evaluating PDMAC_67_-PDAAM_50_ and PNAEP_67_-PS_75_ nanoparticles as putative dispersants. Using the former diblock copolymer led to a significant improvement in milling efficiency: a final particle size of 2.1 μm was achieved after a milling time of just 15 min. The latter diblock copolymer required a milling time of 30 min, which is comparable to the conditions required when using either the PGMA_50_-PMMA_80_ or PGMA_50_-PBzMA_50_ nanoparticles. Clearly, all four types of nanoparticles act as both a wetting agent and an effective dispersant: the chemical nature of the non-ionic stabilizer block has minimal effect on dispersant performance. However, additional experiments were performed using amphiphilic diblock copolymer nanoparticles comprising either cationic poly(2-(methacryloyloxy)ethyl trimethylammonium chloride) [PMETAC] or anionic poly(methacrylic acid) [PMAA] as the steric stabilizer block (Figure S1). Compared to sterically stabilized nanoparticles prepared using non-ionic steric stabilizers, such nanoparticles exhibit comparable DLS diameters (35 and 29 nm, respectively) but strikingly different electrophoretic footprints (Figure S2). However, in neither case was it possible to obtain a final volume-average diameter of 2 μm for azoxystrobin microparticles even after a milling time of 60 min. Moreover, such formulations generated many air bubbles and/or foam, which could not be suppressed by adding an antifoam agent. Thus, polyelectrolytic steric stabilizers do not seem to be appropriate for the design of efficient nanoparticle dispersants, at least in the case of azoxystrobin.

### Effect of Varying the Mean Nanoparticle Diameter

**Figure 3:**
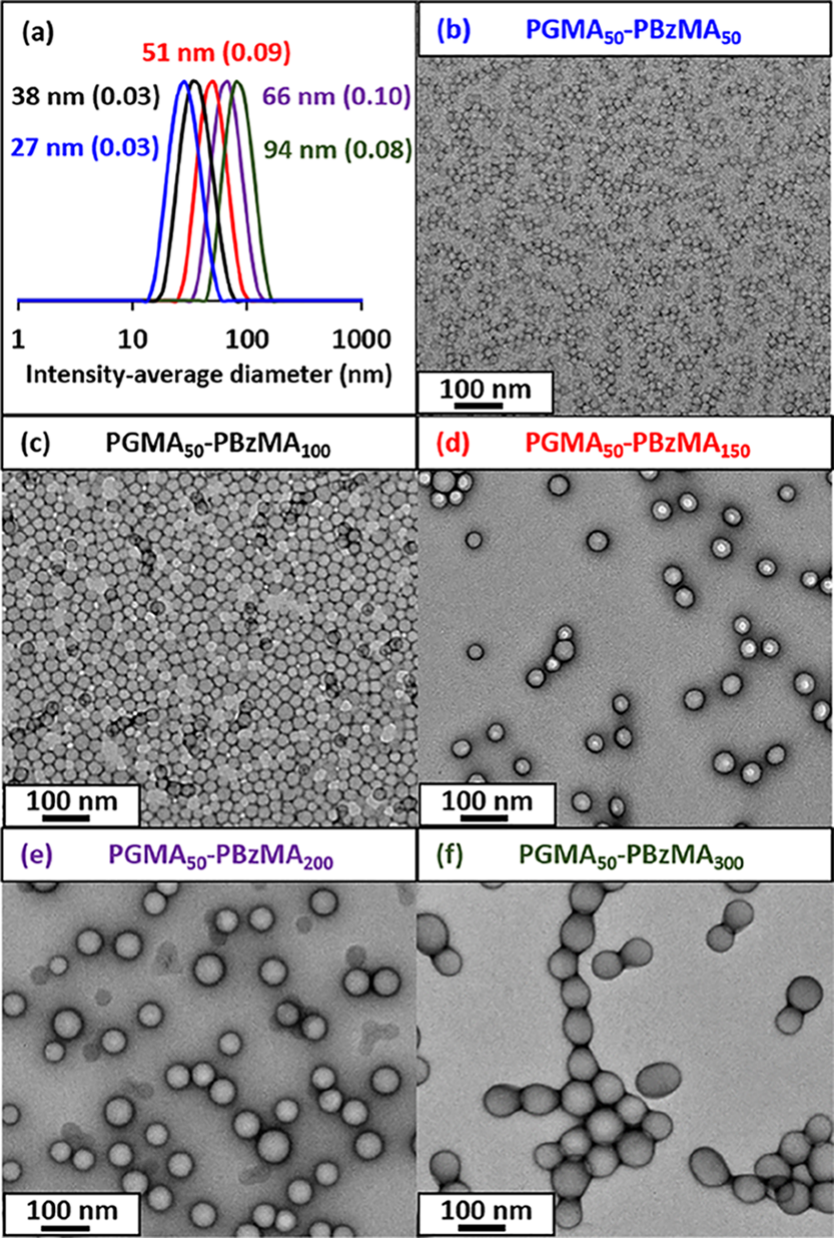
(a) DLS intensity-average particle size distributions recorded (plus *z*-average diameters and DLS polydispersities) for PGMA_50_-PBzMA_*x*_ nanoparticles, where *x* is varied from 50 to 300. (b–f) Corresponding TEM images obtained for the same series of five PGMA_50_-PBzMA_50-300_ nanoparticles prepared via RAFT aqueous emulsion polymerization of BzMA according to Scheme 1.

**Scheme 1:**
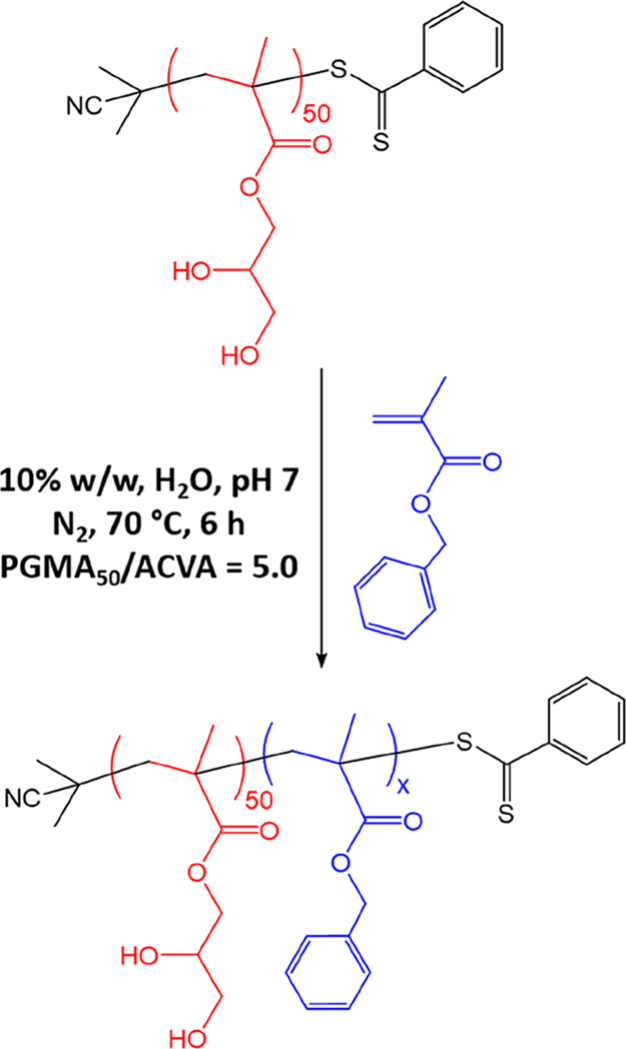
Synthesis of PGMA_50_-PBzMA_*x*_ Diblock Copolymer Nanoparticles by RAFT Aqueous Emulsion Polymerization of BzMA Using a PGMA_50_ Precursor Under the Stated Conditions Systematic variation of the target degree of polymerization of the PBzMA block (*x*) enables the mean nanoparticle diameter to be tuned (see main text for further details).

A series of PGMA_50_-PBzMA_*x*_ nanoparticles were prepared in which the mean diameter was systematically varied simply by increasing the target DP for the core-forming PBzMA block (Scheme 1). More specifically, targeting PBzMA DPs of 50 to 300 led to *z*-average diameters ranging from 27 to 94 nm as judged by DLS (Figure 3). TEM studies indicated an increase in the number-average particle diameter (Figure 3) and confirmed that only kinetically trapped spheres were produced (as opposed to higher-order morphologies such as worms or vesicles). Similar observations were reported by Cunningham and co-workers.^18^

**Figure 4:**
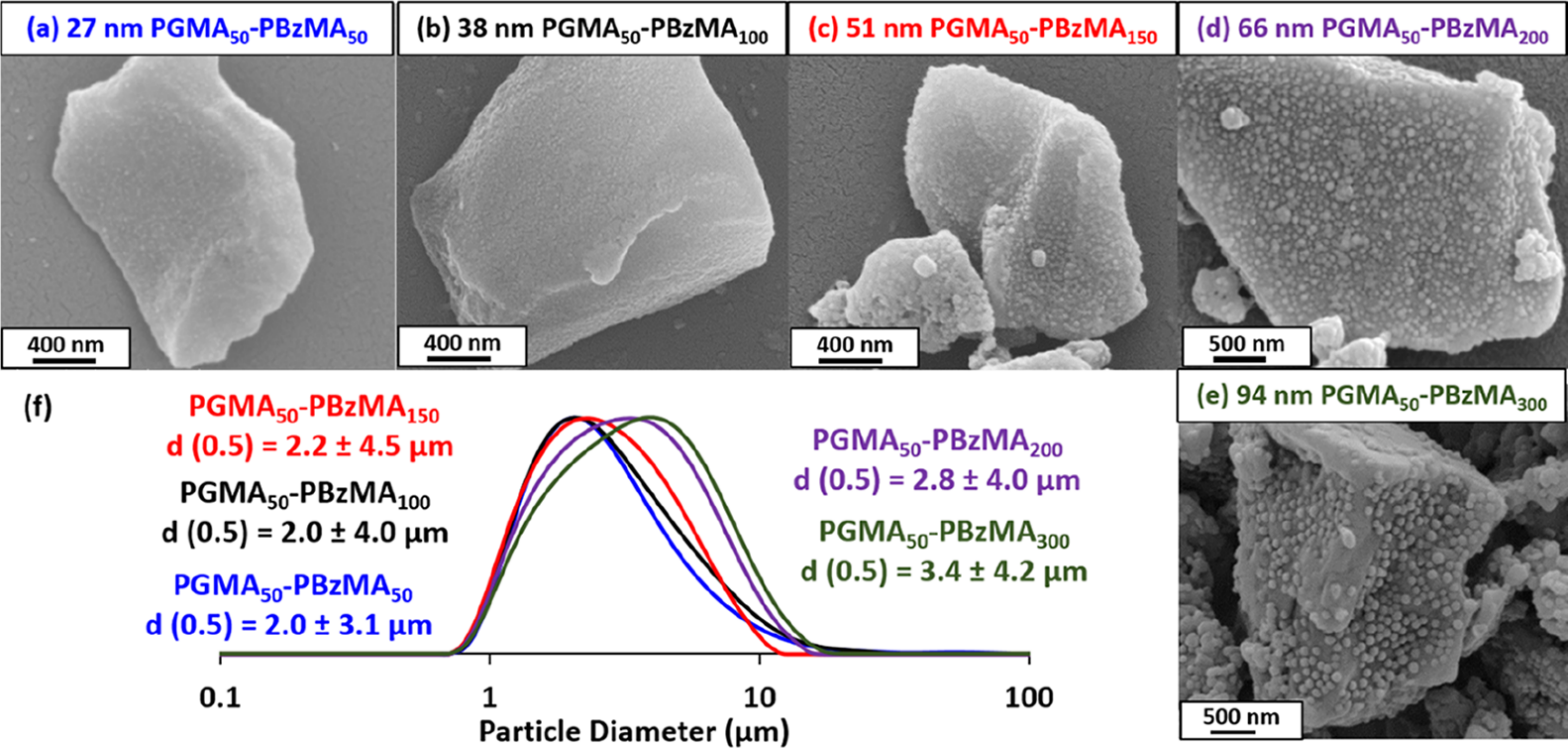
(a–e) SEM images of individual azoxystrobin microparticles prepared via ball milling in the presence of five examples of PGMA_50_-PBzMA_*x*_ nanoparticles of varying size (after removing excess non-adsorbed nanoparticles by centrifugation). (f) Corresponding laser diffraction particle size distribution curves recorded for azoxystrobin microparticles obtained after a milling time of 30 min when using the same five examples of PGMA_50_-PBzMA_*x*_ nanoparticles.

Azoxystrobin was milled in turn using five examples of PGMA_50_-PBzMA_*x*_ nanoparticles of varying *z*-average diameter. In this series of experiments, the dispersant concentration was adjusted to ensure that a constant total surface area of nanoparticles was used to prepare each SC. Full details of these formulations are summarized in Table S2. Laser diffraction was used to size the azoxystrobin microparticles after milling for 30 min (Figure 4). A volume-average particle diameter of approximately 2 μm was obtained when milling azoxystrobin in the presence of PGMA_50_-PBzMA_50_, PGMA_50_-PBzMA_100_ or PGMA_50_-PBzMA_150_ nanoparticles (which possessed *z*-average diameters of 27, 38 or 51 nm, respectively). In contrast, milling for 30 min in the presence of the two largest nanoparticle dispersants (i.e., PGMA_50_-PBzMA_200_ or PGMA_50_-PBzMA_300_) only produced relatively large azoxystrobin microparticles of approximately 3 μm diameter.

Three centrifugation–redispersion cycles were performed on the resulting SCs to remove any non-adsorbed excess nanoparticles. Figure 4 shows SEM images recorded for such purified azoxystrobin microparticles. In each case, individual microparticles are uniformly coated with a layer of adsorbed PGMA_50_-PBzMA_*x*_ nanoparticles. Moreover, using larger nanoparticles appears to result in lower surface coverages. This study suggests that smaller spheres ensure the most efficient milling and perhaps also lead to higher surface coverages, at least when milling azoxystrobin in the presence of this particular class of nanoparticle dispersants. The long-term stability of this series of aqueous SCs was also assessed using laser diffraction (see later).

### Effect of Crosslinking the Nanoparticle Cores

**Figure 5:**
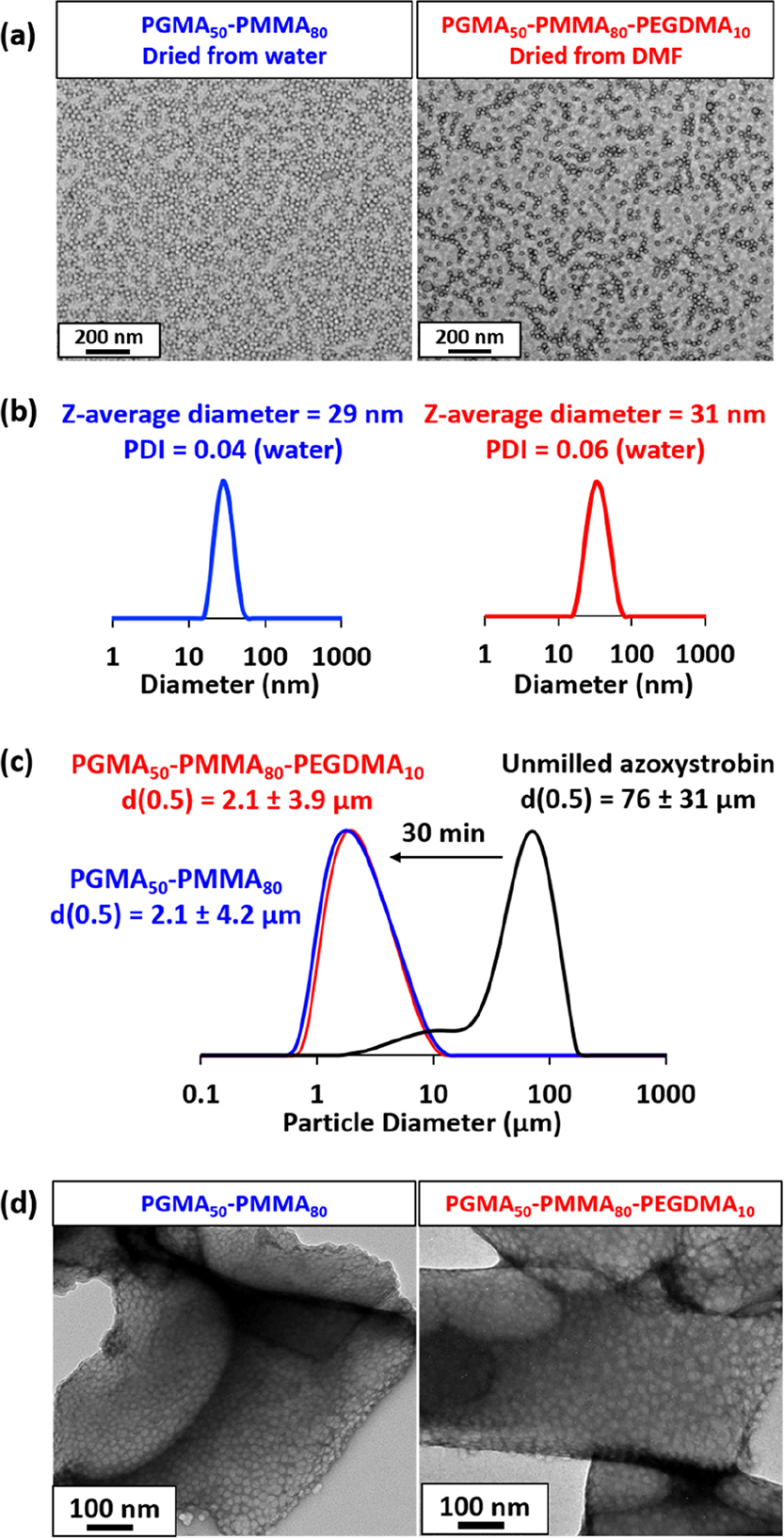
(a) TEM images obtained for linear PGMA_50_-PMMA_80_ nanoparticles dried from water and core-crosslinked PGMA_50_-PMMA_80_-PEGDMA_10_ nanoparticles dried from DMF. (b) DLS intensity-average particle size distributions recorded for 0.1% w/w aqueous dispersions of linear PGMA_50_-PMMA_80_ (blue trace) and core-crosslinked PGMA_50_-PMMA_80_-PEGDMA_10_ nanoparticles (red trace). (c) Laser diffraction particle size distribution curves (and corresponding volume-average diameters) recorded for the unmilled azoxystrobin (black) and milled azoxystrobin coated with either linear PGMA_50_-PMMA_80_ nanoparticles (blue) or core-crosslinked PGMA_50_-PMMA_80_-PEGDMA_10_ nanoparticles (red). (d) TEM images recorded for azoxystrobin microparticles prepared by milling in the presence of either linear or core-crosslinked nanoparticle dispersions after removal of excess non-adsorbed nanoparticles by centrifugation.

In 2012 Chambon et al. reported that linear diblock copolymer nano-objects prepared via aqueous PISA could be covalently stabilized simply by chain extension using a divinyl monomer to generate a third block.^42^ Accordingly, core-crosslinked PGMA_50_-PMMA_80_-PEGDMA_10_ nanoparticles were readily prepared by adding 12.5 mol % EGDMA (based on MMA monomer) after the MMA was fully consumed (Scheme S1). Representative TEM images obtained for the linear PGMA_50_-PMMA_80_ precursor nanoparticles dried from water and the final core-crosslinked PGMA_50_-PMMA_80_-PEGDMA_10_ nanoparticles dried from DMF are shown in Figure 5a. The former nanoparticles exhibit a well-defined spherical morphology, as expected. DMF is a good solvent for both the PGMA_50_ stabilizer block and the PMMA_80_ core-forming block; thus, molecular dissolution of the linear nanoparticles occurs in this solvent (indeed, DMF is the eluent of choice for GPC analysis of such diblock copolymer chains).^40^ However, TEM indicates a similar spherical morphology for the PGMA_50_-PMMA_80_-PEGDMA_10_ nanoparticles dried from DMF, which confirms successful core-crosslinking in this case. Moreover, DLS studies of the same PGMA_50_-PMMA_80_-PEGDMA_10_ nanoparticles dispersed in DMF (data not shown) indicated the presence of slightly swollen spheres with a *z*-average diameter of 34 nm, rather than molecularly dissolved copolymer chains. Given that the linear precursor PGMA_50_-PMMA_80_ nanoparticles had a *z*-average diameter of 29 nm, this suggests a relatively high degree of core crosslinking. Furthermore, DLS experiments conducted on a dilute *aqueous* dispersion of the PGMA_50_-PMMA_80_-PEGDMA_10_ nanoparticles indicated a *z*-average particle diameter of 31 nm (Figure 5b), which suggests that core crosslinking has minimal effect on the nanoparticle dimensions.

Subsequently, the nanoparticle dispersant performance of the core-crosslinked nanoparticles was directly compared to that of the linear nanoparticles for the same SC formulation under identical milling conditions. The SCs produced in each case were then sized by laser diffraction (Figure 5c). Clearly, covalent stabilization of the nanoparticle cores has essentially no effect on the size of the final azoxystrobin microparticles. This is an important observation because it eliminates the possibility that individual amphiphilic diblock copolymer chains are in equilibrium with the linear diblock copolymer nanoparticles, with the former species potentially playing an important role in either initial surface wetting or subsequent steric stabilization of the azoxystrobin microparticles.

Moreover, three centrifugation–redispersion cycles were performed to remove any excess non-adsorbed nanoparticles from these two SCs. TEM images of the resulting purified azoxystrobin microparticles are shown in Figure 5d. A relatively high surface coverage is obtained when using either the linear PGMA_50_-PMMA_80_ nanoparticles or the core-crosslinked PGMA_50_-PMMA_80_-PEGDMA_10_ nanoparticles. Such images provide compelling evidence that crosslinking the nanoparticle cores has no discernible effect on either the milling efficiency or their ability to adsorb at the surface of the azoxystrobin microparticles.

### Effect of Varying the Glass Transition Temperature (*T*_g_) of the Core-Forming Block

High-*T*_g_ PNAEP_67_-PS_100_ nanoparticles were prepared by RAFT aqueous emulsion polymerization of styrene.^33^ In addition, analogous diblock copolymer nanoparticles comprising a core-forming statistical block exhibiting a much lower *T*_g_ were prepared by statistical copolymerization of styrene (45 wt %) with *n*-butyl acrylate (55 wt %) using the same PNAEP_67_ precursor.^33^ Differential scanning calorimetry (DSC) curves recorded for the PNAEP_67_ precursor, PNAEP_67_-PS_100_ nanoparticles, and PNAEP_67_-P(S-*stat*-nBA)_100_ nanoparticles are shown in Figure S3. The PNAEP_67_-PS_100_ diblock copolymer exhibits two *T*_g_ values at −1.8 and 83.4 °C, respectively, which are the results of microphase separation between the two mutually incompatible blocks. In contrast, only a single *T*_g_ of 8.6 °C was observed for the PNAEP_67_-P(S-*stat*-nBA)_100_ diblock copolymer.

**Figure 6:**
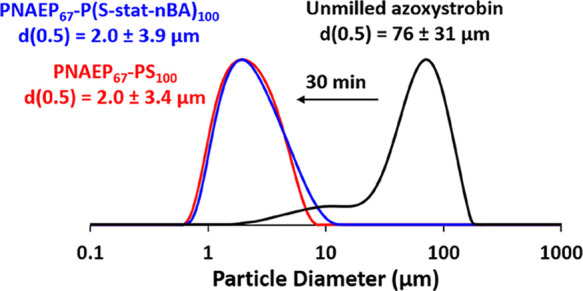
Laser diffraction particle size distribution curves (and corresponding volume-average diameters) recorded after milling azoxystrobin with either PNAEP_67_-PS_100_ nanoparticles (red curve) or PNAEP_67_-P(S-*stat*-nBA)_100_ nanoparticles (blue curve) for 30 min.

DLS studies indicated that these PNAEP_67_-PS_100_ and PNAEP_67_-P(S-*stat*-nBA)_100_ nanoparticles had comparable *z*-average particle diameters of 35 and 39 nm, respectively (Figure S4). Both types of nanoparticles were evaluated as putative dispersants during the milling of azoxystrobin. Laser diffraction studies confirmed that azoxystrobin microparticles with a volume-average diameter of approximately 2 μm could be obtained after milling for 30 min when using either nanoparticle dispersant (Figure 6). SEM images of the azoxystrobin microparticles recorded after the removal of excess nanoparticles are shown in Figure S5. These experiments suggest that retention of the original copolymer morphology is not required for sterically stabilized nanoparticles to act as a dispersant for azoxystrobin.

### Effect of Varying the Chemical Nature of the Agrochemical Active

We sought to establish whether this nanoparticle dispersant approach was also applicable to alternative hydrophobic organic crystalline compounds exhibiting minimal aqueous solubility. Accordingly, the following five agrochemical actives were evaluated for the preparation of nanoparticle-stabilized aqueous SCs: CCZ, DFZ, IZM, TEB and PXD (Figure 1a). The first four compounds are alternative fungicides to azoxystrobin with varying modes of action, whereas the latter is a highly selective systemic herbicide that is used to control monocotyledonous grass weeds in crops such as wild oats, wheat and barley.^43−46^

**Figure 7:**
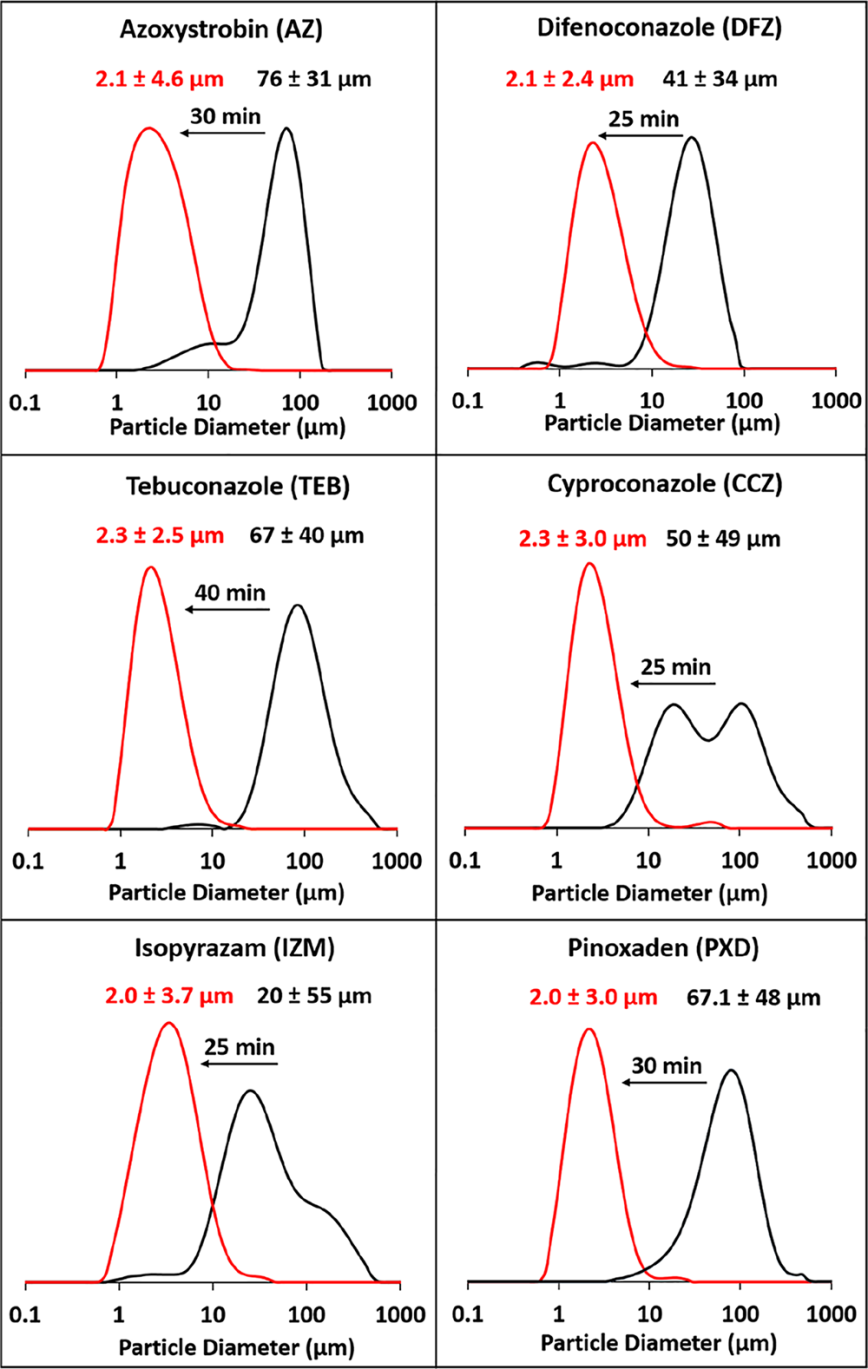
Laser diffraction particle size distribution curves (and corresponding volume-average diameters) recorded for (i) six unmilled (black curves) agrochemical AIs (azoxystrobin, DFZ, TEB, CCZ, IZM and PXD) and (ii) after milling each of these AIs in the presence of PGMA_50_-PMMA_80_ nanoparticles (red curves).

PGMA_50_-PMMA_80_ nanoparticles were used as the dispersant when attempting to mill each of these five agrochemicals. SC formulations comprising just the agrochemical active, the nanoparticle dispersant, an antifoam agent, and water were used in this set of experiments. Figure 7 summarizes the laser diffraction curves recorded before and after milling: organic microparticles with a volume-average particle diameter of approximately 2 μm could be obtained in each case after milling for 25–40 min using the IKA tube drive. Optical microscopy images recorded for (i) the various coarse crystals prior to milling and (ii) the much finer corresponding microparticles obtained after milling are shown in Figure S6. These observations clearly demonstrate that PGMA_50_-PMMA_80_ nanoparticles can act as an effective wetting agent and dispersant for a range of agrochemical actives, not just azoxystrobin.

**Figure 8:**
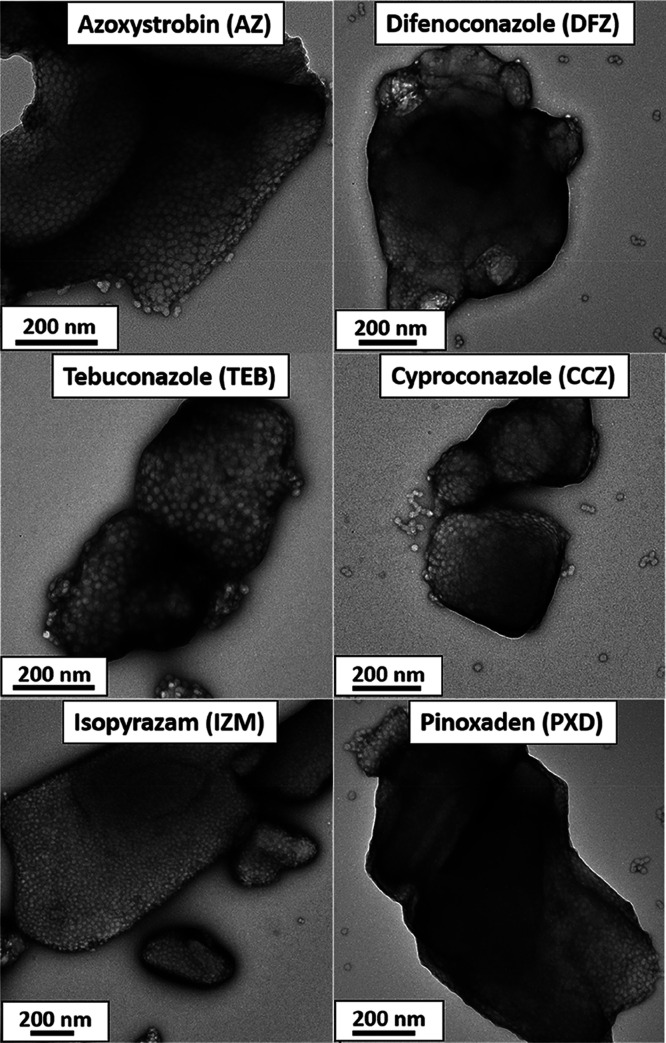
TEM images recorded for microparticles prepared by milling six different agrochemical AIs in the presence of PGMA_50_-PMMA_80_ nanoparticles (after removal of excess nanoparticles by centrifugation–redispersion cycles). In each case, the nanoparticles are clearly adsorbed at the surface of the organic crystalline microparticles at relatively high surface coverage.

These five new SCs were each subjected to three centrifugation–redispersion cycles to remove any non-adsorbed PGMA_50_-PMMA_80_ nanoparticles. Figure 8 shows representative TEM images of individual CCZ, DFZ, IZM, TEB and PXD microparticles, which are each coated with a uniform layer of PGMA_50_-PMMA_80_ nanoparticles. For the IZM microparticles, digital image analysis using ImageJ software indicates a surface coverage of approximately 40–45%. At first sight, this is significantly higher than that estimated by XPS studies for azoxystrobin microparticles coated with the same nanoparticles (24% surface coverage).^39^ However, we found that the grayscale adjustment within ImageJ software is rather subjective, so this relatively high fractional surface coverage ideally requires corroboration by XPS. Unfortunately, this is beyond the scope of the current study.

In summary, nanoparticle adsorption onto micrometer-sized organic crystalline agrochemical particles appears to be a rather general phenomenon. It occurs regardless of the type of nanoparticle core and is observed for several types of non-ionic steric stabilizers and six agrochemical actives. However, such adsorption does not seem to involve any electrostatic component because neither cationic nor anionic steric stabilizers promote nanoparticle adsorption. The adsorption of *soluble* polymer chains onto surfaces is a rather generic enthalpically driven phenomenon;^47^ the same appears to be true for (non-ionic) sterically stabilized nanoparticles.

### Long-Term Stability of Azoxystrobin-Based SCs

**Figure 9:**
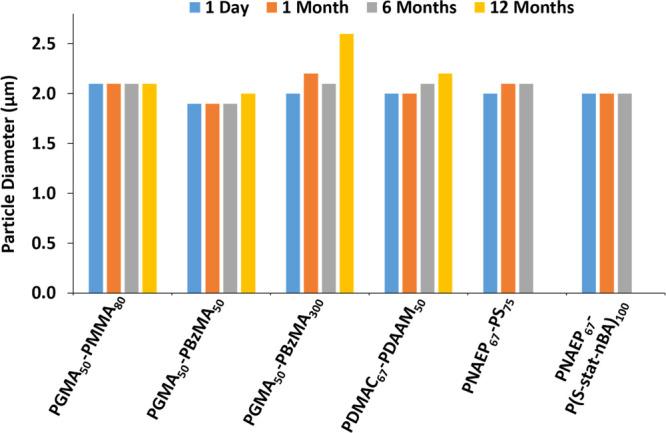
Volume-average particle diameter data obtained via laser diffraction for various azoxystrobin-based suspension concentrates using the stated diblock copolymer nanoparticles as dispersants after ageing at 20 °C for 1 day, 1 month, 6 months, or 12 months. In such experiments, an approximately constant mean particle diameter indicates a stable suspension concentrate.

The long-term stability of azoxystrobin-based SCs was assessed using laser diffraction. Given the mean size and density of the azoxystrobin microparticles, such formulations tended to sediment over time in the absence of any structuring agents. However, in each case, redispersion was readily achieved upon hand-shaking. This enabled particle size analysis to be conducted on each suspension after 1, 6 and 12 months, as well as on the fresh (i.e., day-old) suspension (Figure 9).

In each case, the original SC exhibited an initial volume-average particle diameter of approximately 2 μm after ball milling. For the formulation prepared using the largest PGMA_50_-PBzMA_300_ nanoparticles, the milling time was extended to 45 min to achieve the desired 2 μm diameter for the azoxystrobin microparticles. These SCs exhibited minimal change in the particle size after 6 months and, in most cases, remained stable after 1 year of storage at ambient temperature. The outlier was the SC prepared using the largest PGMA_50_-PBzMA_300_ nanoparticles, but even for this least stable formulation, the mean particle diameter only increased from 2.0 to 2.5 μm after 12 months. Interestingly, there was no discernible difference in long-term stability when varying the chemical nature of the steric stabilizer block, the core-forming block, or when employing soft, film-forming nanoparticles as the dispersant.

## Conclusions

Various sterically stabilized diblock copolymer nanoparticles prepared via RAFT polymerization using various aqueous PISA formulations are shown to be effective dispersants for the preparation of SCs comprising six different agrochemical actives via wet ball milling. Changing the chemical nature of the non-ionic core-forming block had essentially no effect on the dispersant performance. However, nanoparticles comprising either cationic or anionic steric stabilizer chains proved to be ineffective. A series of PGMA_50_-PBzMA_*x*_ nanoparticles with varying mean diameters were also evaluated as dispersants. In this case, nanoparticles of up to 51 nm diameter were effective, but larger nanoparticles led to less efficient ball milling and the formation of marginally less stable microparticles. The effect of (i) crosslinking the nanoparticle cores and (ii) lowering the *T*_g_ of the core-forming block was also examined. In the former case, the covalently stabilized nanoparticles performed as well as the corresponding linear nanoparticles, which suggests that individual amphiphilic diblock copolymer chains do not play a significant role in the production of SCs. In the latter case, stable SCs could be obtained when using film-forming nanoparticles, so preservation of the original copolymer morphology after adsorption at the surface of the azoxystrobin crystals is not a prerequisite for successful processing. Moreover, this nanoparticle dispersant approach developed for azoxystrobin was extended to include five other widely used agrochemical actives with various physicochemical properties, which suggests that it is likely to be generic in scope. Finally, preliminary long-term stability studies of azoxystrobin-based SCs using laser diffraction suggest that most of these formulations remained stable for at least 1 year.

## Supporting Information Available

Full experimental details for all PISA formulations and analytical techniques used in this study; tabulated summary of the chemical structures and physicochemical properties for the six agrochemical actives; TEM and DLS studies for PMETAC_46_-PMMA_50_ and PMAA_56_-PMMA_50_ diblock copolymer nanoparticles; laser diffraction data recorded for unmilled azoxystrobin and milled azoxystrobin in the presence of either PMETAC_46_-PMMA_50_ or PMAA_56_-PMMA_50_ nanoparticles; aqueous electrophoresis data recorded for PMETAC_46_-PMMA_50_, PGMA_50_-PMMA_80_, and PMAA_56_-PMMA_50_ diblock copolymers; summary table for SC formulations when using PGMA_50_-PBzMA_*x*_ nanoparticles of varying size; schematic synthesis of PGMA_50_-PMMA_80_-PEGDMA_10_ triblock copolymer nanoparticles; DSC data recorded for PNAEP_67_-PS_100_, PNAEP_67_-P(S-stat-nBA)_100_, and the PNAEP_67_ precursor; DLS data recorded PNAEP_67_-PS_100_ and PNAEP_67_-P(S-stat-nBA)_100_ diblock copolymers; SEM images recorded for milled azoxystrobin in the presence of either PNAEP_67_-PS_100_ or PNAEP_67_-P(S-stat-nBA)_100_ diblock copolymers; and optical microscopy images recorded for unmilled AI crystals and the corresponding microparticles obtained after milling in the presence of PGMA_50_-PMMA_80_ nanoparticles (PDF)
