# Supplementary material for: Sterically Stabilized Diblock Copolymer Nanoparticles Enable Convenient Preparation of Suspension Concentrates Comprising Various Agrochemical Actives
Source: Langmuir. 2022 Feb 22;38(9):2885–94. doi: 10.1021/acs.langmuir.1c03275 (PMC9007534; doi:10.1021/acs.langmuir.1c03275)
Supplement: Supplementary file 1 — la1c03275_si_001.pdf [file la1c03275_si_001.pdf]

Sterically-Stabilized Diblock Copolymer Nanoparticles Enable Convenient Preparation of Suspension Concentrates Comprising Various Agrochemical Actives

Derek H. H. Chan,[a] Oliver J. Deane,[a] Emily L. Kynaston,[b] Christopher Lindsay,[b] Philip Taylor[b] and Steven P. Armes[a]*

a. Dainton Building, Chemistry Department, University of Sheffield, Brook Hill, Sheffield, South Yorkshire, S3 7HF, UK.

b. Syngenta, Jealott's Hill International Research Centre, Bracknell, Berkshire, RG42 6EY, UK.

Summary of Contents

Experimental section for all PISA syntheses and characterization techniques

Table S1. Summary of chemical structures and physicochemical properties for the six agrochemical active ingredients used in this study.

Figure S1. Chemical structures, TEM and DLS data for $PMETAC_{46}$-$PMMA_{50}$ and $PMAA_{56}$-$PMMA_{50}$ nanoparticles. Laser diffraction particle size distribution curves obtained before and after milling of azoxystrobin when using such nanoparticles.

Figure S2. Aqueous electrophoresis data recorded for $PMETAC_{46}$-$PMMA_{50}$, $PGMA_{50}$-$PMMA_{80}$ and $PMAA_{56}$-$PMMA_{50}$ nanoparticles.

Table S2. Suspension concentrate formulations to evaluate the effect of varying the mean diameter of a series of $PGMA_{50}$-$PBzMA_x$ nanoparticles.

Scheme S1. Schematic synthesis of core-crosslinked $PGMA_{50}$-$PMMA_{80}$-$PEGDMA_{10}$ triblock copolymer nanoparticles via sequential RAFT aqueous emulsion polymerization of (i) MMA and (ii) EGDMA at 70 °C.

Figure S3. Differential scanning calorimetry curves recorded for dried $PNAEP_{67}$-PS_{100} nanoparticles, dried $PNAEP_{67}$-P(S-*stat*-nBA)$_{100}$ nanoparticles (nBA = 55% by mass) and the $PNAEP_{67}$ precursor.

Figure S4. DLS data obtained for $PNAEP_{67}$-PS_{100} and $PNAEP_{67}$-P(S-*stat*-nBA)$_{100}$ nanoparticles.

Figure S5. SEM images recorded for azoxystrobin microparticles prepared using $PNAEP_{67}$-PS_{100} or $PNAEP_{67}$-P(S-*stat*-nBA)$_{100}$ nanoparticles.

Figure S6. Optical microscopy images recorded for five agrochemical active ingredients (difenoconazole, tebuconazole, cyproconazole, isopyrazam and pinoxaden) before and after milling.

Experimental

Materials

Methyl methacrylate (MMA, 99%), 4,4′-azobis(4-cyanovaleric acid) (ACVA, 98%), 2-cyano-2-propyl benzodithioate (CPDB, 97%), *N,N*-dimethylacrylamide (DMAC, 99%), benzyl methacrylate (BzMA, 98%), styrene (S, 99%), *n*-butyl acrylate (nBA, 99%) and ethylene glycol dimethylacrylate (EGDMA, 98%) were purchased from Sigma-Aldrich (Gillingham, UK). Diacetone acrylamide (DAAM, 99%) was purchased from Alfa Aesar (UK) while methyl-2 (dodecylthiocarbonothioylthio)-2-methylpropionate (Me-DDMAT) was prepared according to a literature protocol.[41] Glycerol monomethacrylate (GMA, 99.8%) was donated by GEO Specialty Chemicals (Hythe, UK). 2-(*N*-Acryloyloxy)ethyl pyrrolidone (NAEP; 95%) was provided by Ashland Specialty Ingredients (Cherry Hill, NJ, USA). Azoxystrobin (AZ), tebuconazole (TEB), difenoconazole (DFZ), cyproconazole (CCZ), isopyrazam (IZM) and pinoxaden (PXD) were provided by Syngenta (Jealott's Hill, UK). 1.0 mm zirconium aluminium oxide beads were purchased from Sigmund-Lindner (Germany). Silicone SAG1572 antifoam was purchased from Momentive (Germany). Deionized water was used for all experiments.

Synthesis of $PGMA_{50}$ Precursor by RAFT Aqueous Solution Polymerization of GMA

GMA monomer (30.0 g, 187 mmol), CPDB (0.589 g, 2.66 mmol; target degree of polymerization, DP, for PGMA = 70), ACVA initiator (0.149 g, 0.53 mmol; CPDB/ACVA molar ratio = 5.0), and ethanol (46.5 g, 60% w/w) were weighed into a 250 mL round-bottom flask. This flask was placed in an ice bath and the ethanolic solution was deoxygenated using a N_2 sparge for 30 min. The flask was then immersed in an oil bath set at 70 °C for 165 min and a final GMA conversion of 71% was determined by ^{1}H NMR spectroscopy. The crude polymer solution was diluted with methanol (30 mL) and then precipitated into a ten-fold excess of dichloromethane (twice). ^{1}H NMR spectroscopy studies indicated a mean DP of 50 via end-group analysis (the integrated aromatic signals at 7.4 to 7.8 ppm were compared to that of the methacrylic backbone at 0.7 to 2.5 ppm).

Synthesis of $PGMA_{50}$-$PBzMA_x$ Diblock Copolymer Nanoparticles by RAFT Aqueous Emulsion Polymerization

A typical synthesis of $PGMA_{50}$-$PBzMA_x$ nanoparticles was conducted according to the following protocol. $PGMA_{50}$ precursor (0.150 g, 18.2 µmol), BzMA monomer (0.161 g, 0.91 mmol; target DP = 50), ACVA initiator (1.0 mg, 3.65 µmol; $PGMA_{50}$/ACVA molar ratio = 5.0) and deionized water (2.806 g, 10 % w/w solution) were added to a 20 mL round-bottom flask. This flask was placed in an ice bath

and the resulting aqueous solution was deoxygenated using a N_2 sparge for 30 min. The flask was then immersed in an oil bath set at 70 °C for 6 h. The BzMA polymerization was quenched by exposing the contents of the flask to air while cooling to 20 °C.

Synthesis of Linear $PGMA_{50}$-$PMMA_{80}$ Diblock Copolymer and Cross-linked $PGMA_{50}$-$PMMA_{80}$-$PEGDMA_{10}$ Triblock Copolymer Nanoparticles by RAFT Aqueous Emulsion Polymerization

The synthesis of linear $PGMA_{50}$-$PMMA_{80}$ nanoparticles by RAFT aqueous emulsion polymerization was conducted as follows. $PGMA_{50}$ precursor (0.150 g, 18.2 µmol), MMA monomer (0.146 g, 1.46 mmol; target DP = 80), ACVA initiator (1.0 mg, 3.64 µmol; $PGMA_{50}$/ACVA molar ratio = 5.0) and deionized water (2.675 g, 10 % w/w solution) were added to a 20 mL round-bottom flask. The flask was placed in an ice bath and the aqueous emulsion was deoxygenated using a N_2 sparge for 30 min and then immersed in an oil bath set at 70 °C for 3 h. The polymerization was quenched by exposing the contents of the flask to air while cooling to 20 °C. Cross-linked nanoparticles were prepared from these linear nanoparticles by adding EGDMA (0.036 g, 0.182 mmol; target DP = 10) to grow a third block. The aqueous dispersion was stirred for 12 h at 70 °C to ensure full EGDMA conversion and the polymerization was quenched by exposing the contents of the flask to air while cooling to 20 °C.

Synthesis of $PDMAC_{67}$-$PDAAM_{50}$ Diblock Copolymer Nanoparticles by RAFT Aqueous Dispersion Polymerization

The poly(*N,N*-dimethylacrylamide) $PDMAC_{67}$ precursor was prepared via RAFT solution polymerization using a literature protocol.[41] The synthesis of $PDMAC_{67}$-$PDAAM_{50}$ nanoparticles by RAFT aqueous dispersion polymerization was conducted as follows. $PDMAC_{67}$ precursor (0.200 g, 28.4 µmol), DAAM monomer (0.241 g, 1.42 mmol; target DP = 50) and ACVA initiator (0.80 mg, 2.85 µmol; $PDMAC_{67}$/ACVA molar ratio = 10) were weighed into a 20 mL round-bottom flask. Deionized water (1.767 g) was adjusted to pH 2.5 using HCl and added to make up a 20% w/w solution. The flask was placed in an ice bath and the aqueous solution was deoxygenated using a N_2 sparge for 15 min prior to immersing the flask in an oil bath set at 70 °C. After 4 h, the polymerization was quenched by exposing the contents of the flask to air while cooling to 20 °C.

Synthesis of PNAEP$_{67}$, PNAEP$_{67}$-PS$_{75}$ diblock copolymer nanoparticles and PNAEP$_{67}$ P(S-stat-nBA)$_{100}$ diblock copolymer nanoparticles by RAFT Aqueous Emulsion Polymerization

The synthesis of the poly(2-(*N*-acryloyloxy)ethyl pyrrolidone) (PNAEP) precursor by RAFT aqueous solution polymerization and the preparation of both PNAEP$_{67}$-PS$_{75}$ and PNAEP$_{67}$ P(S-*stat*-PnBA)$_{100}$ diblock copolymer nanoparticles by RAFT aqueous emulsion polymerization have been previously reported by Deane and co-workers.[33] The same protocols were adopted in the present study.

Preparation of Suspension Concentrates by Ball Milling

A typical protocol used for the preparation of a suspension concentrate (SC) was as follows. Azoxystrobin (2.00 g), PGMA$_{50}$-PBzMA$_{50}$ nanoparticles (0.25 g, 2.5% w/w), SAG1572 antifoam (0.10 g, 1.0% w/w) and deionized water (7.65 g) were added to a 30 mL tube containing 1.0 mm ceramic beads (10.0 g). An IKA Ultra-Turrax Tube Drive was used to mill this suspension at 6 000 rpm for 15-40 min. The beads were removed by filtration to afford a 20% w/w suspension concentrate. The SC had an initial (ambient) temperature of 20 °C and the final temperature was 32 °C at the end of milling.

Centrifugal Purification of Suspension Concentrates

SCs were purified by centrifugation using a Thermo Heraeus Biofuge Pico centrifuge for 5 min at 5 000 rpm. The aqueous supernatant was carefully decanted and the sedimented microparticles were redispersed using deionized water. Two further centrifugation-redispersion cycles were performed before characterization to ensure removal of any excess non-adsorbed nanoparticles.

Characterization Techniques

Dynamic Light Scattering and Aqueous Electrophoresis

A Malvern Zetasizer NanoZS instrument was used to perform both DLS and aqueous electrophoresis studies on 0.10% w/w nanoparticle dispersions. Hydrodynamic z-average diameters were determined at 20 °C at a scattering angle of 173° and measurements were averaged over three runs. Aqueous electrophoresis experiments were conducted in the presence of 1 mM KCl as background electrolyte. The pH was adjusted as required with either HCl or NaOH. The Smoluchkowski

approximation was used to calculate zeta potentials (also averaged over three measurements) via the Henry equation.

Optical Microscopy

A Cole-Palmer optical microscope fitted with a Moticam camera and an LCD tablet was used for imaging both the initial unmilled AIs and the final milled AI microparticles.

Transmission Electron Microscopy

Copper/palladium TEM grids (Agar Scientific, UK) were coated with a thin film of amorphous carbon and then treated with a plasma glow discharge for 30 s. Either an aqueous dispersion of nanoparticles or a suspension concentrate (10 µL, 0.10% w/w) was placed on each grid for 60 s before blotting to remove excess sample. Each grid was stained using uranyl formate (9.0 µL of a 0.75 % w/w solution) for 20 s before removing excess stain and drying under vacuum. Grid were imaged at 100 kV using a Philips CM100 TEM instrument equipped with a Gatan 1 k CCD camera.

Laser Diffraction

The initial coarse active ingredients and final milled microparticles were sized by laser diffraction using a Malvern Mastersizer 3000 instrument equipped with a Hydro EV wet dispersion unit set at 2 000 rpm, a red HeNe laser (λ = 633 nm) and a light-emitting blue light source (λ = 470 nm). The volume-average particle diameter, d(0.5), was calculated by averaging over five measurements. After each measurement, the instrument was thoroughly rinsed with deionized water (three times) to prevent contamination.

Scanning Electron Microscopy

SEM Images were recorded using an FEI Inspect-F instrument using an accelerating voltage of 10 kV and a beam current of 200 nA. Samples were dried onto thin glass slides and then sputter-coated with a thin overlayer of gold prior to imaging.

Table S1. Summary of the chemical structures and physicochemical properties for the six agrochemical active ingredients used in this study.

	**Chemical Structure**	**Molecular Weight (g/mol)**	**Melting Point (°C)**	**Aqueous Solubility (ppm at 20 °C)**	**Density (g cm^{-3})**	**log *P***
Azoxystrobin (AZ)	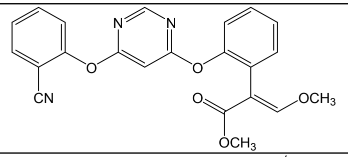	403	116	6.7	1.34	2.5
Difenoconazole (DFZ)	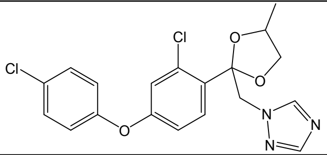	406	79	15	1.39	1.1
Cyproconazole (CCZ)	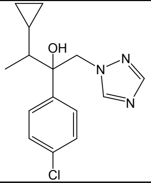	292	106	93	1.26	3.09
Tebuconazole (TEB)	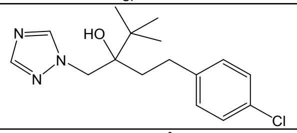	308	105	36	1.25	3.7
Isopyrazam (IZM)	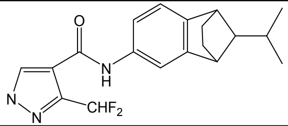	359	127	0.6	1.33	4.25
Pinoxaden (PXD)	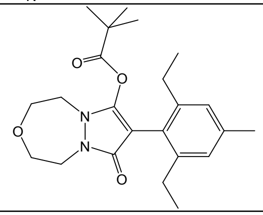	401	121	200	1.16	3.2

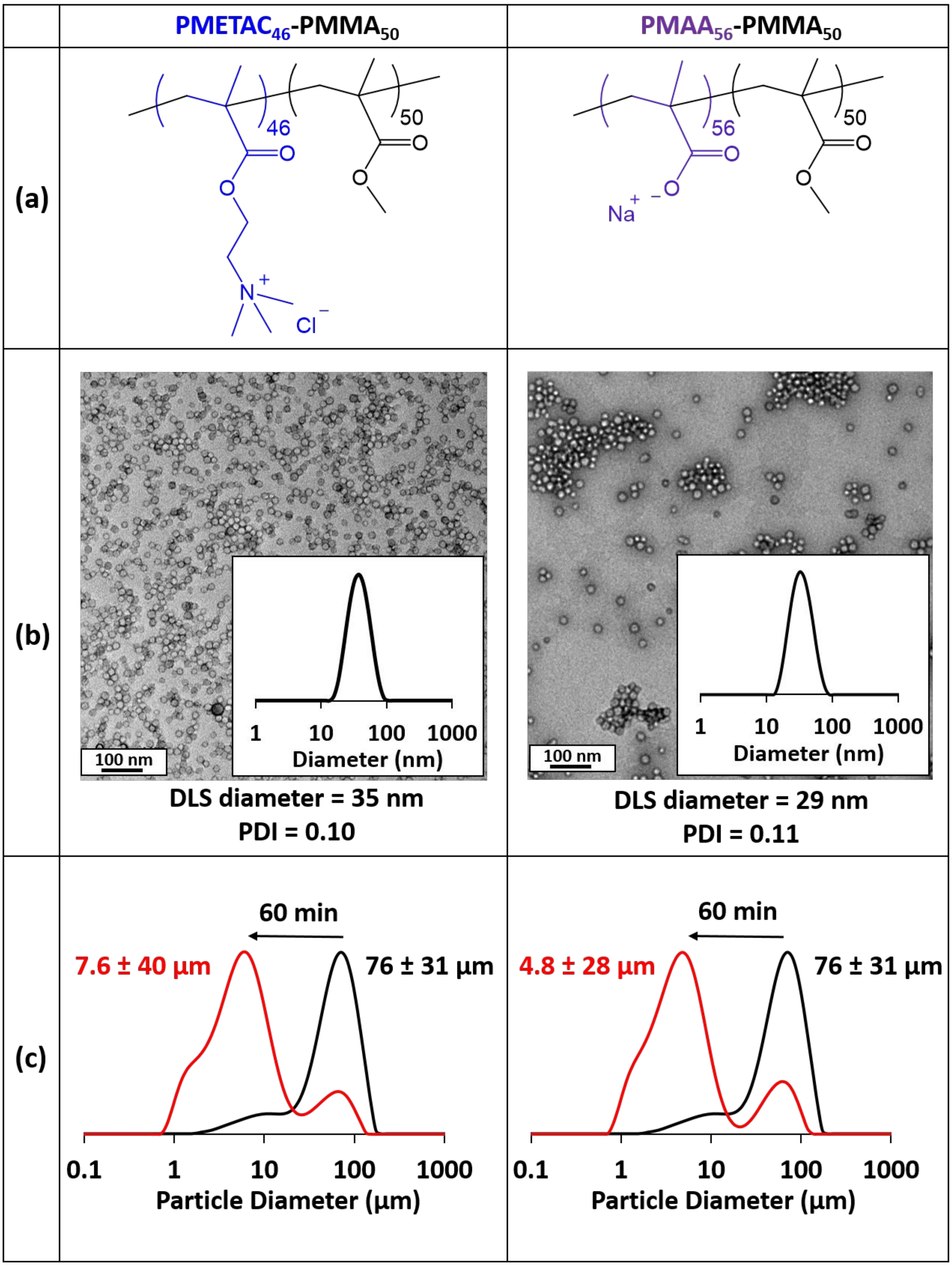

Figure S1. (a) Chemical structure, (b) representative TEM image and DLS intensity-average particle size distribution (see inset) for PMETAC$_{46}$-PMMA$_{50}$ and PMAA$_{56}$-PMMA$_{50}$ nanoparticles; (c) Laser diffraction particle size distributions curves (and corresponding volume-average diameters) recorded for unmilled azoxystrobin (black) and milled azoxystrobin (red) in the presence of either (left) PMETAC$_{46}$-PMMA$_{50}$ or (right) PMAA$_{56}$-PMMA$_{50}$ nanoparticles.

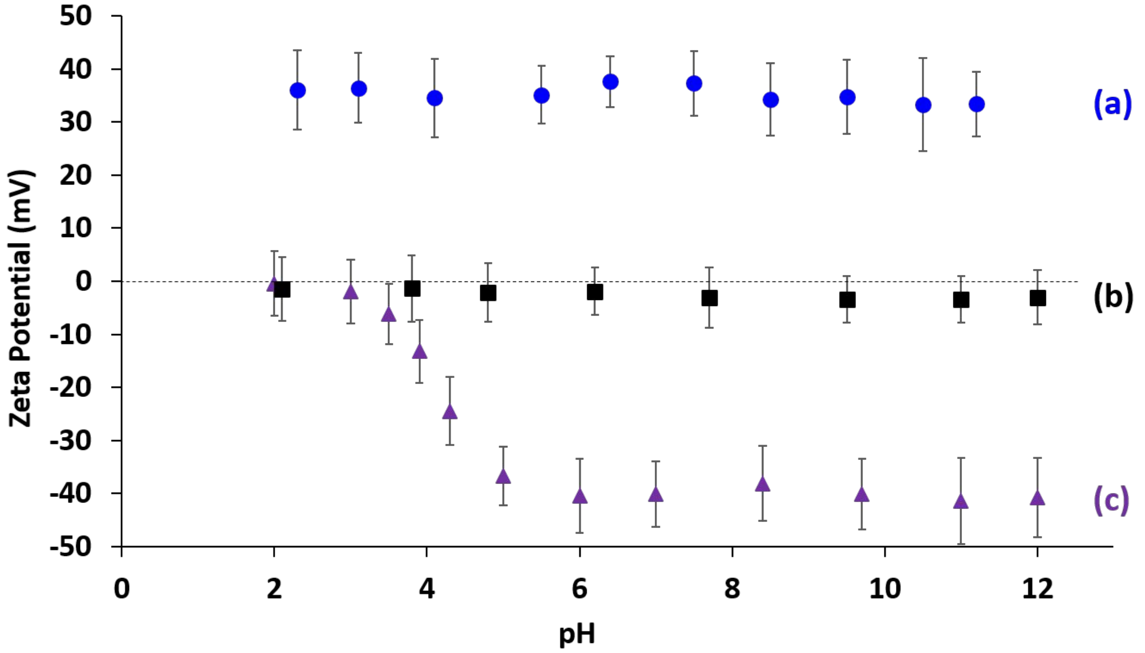

Figure S2. Zeta potential vs pH curves obtained for dilute aqueous dispersions of: (a) $PMETAC_{46}$-$PMMA_{50}$ nanoparticles; (b) $PGMA_{50}$-$PMMA_{80}$ nanoparticles; and (c) $PMAA_{56}$-$PMMA_{50}$ nanoparticles.

Table S2. Summary of suspension concentrate formulations used to evaluate the effect of varying the mean diameter of a series of $PGMA_{50}$-$PBzMA_x$ nanoparticles when milling a fixed mass of azoxystrobin. The mass of nanoparticle dispersant was varied to maintain a constant total surface area (55.6 m^2).

	$PGMA_{50}$-$PBzMA_{50}$	$PGMA_{50}$-$PBzMA_{100}$	$PGMA_{50}$-$PBzMA_{150}$	$PGMA_{50}$-$PBzMA_{200}$	$PGMA_{50}$-$PBzMA_{300}$
**Nanoparticle diameter**	**27 nm**	**38 nm**	**51 nm**	**66 nm**	**94 nm**
**Azoxystrobin**	**2.00 g**	**2.00 g**	**2.00 g**	**2.00 g**	**2.00 g**
**Dispersant**	**0.25 g**	**0.35 g**	**0.47 g**	**0.61 g**	**0.87 g**
**Antifoam**	**0.10 g**	**0.10 g**	**0.10 g**	**0.10 g**	**0.10 g**
**Water**	**7.65 g**	**7.55 g**	**7.43 g**	**7.29 g**	**7.03 g**

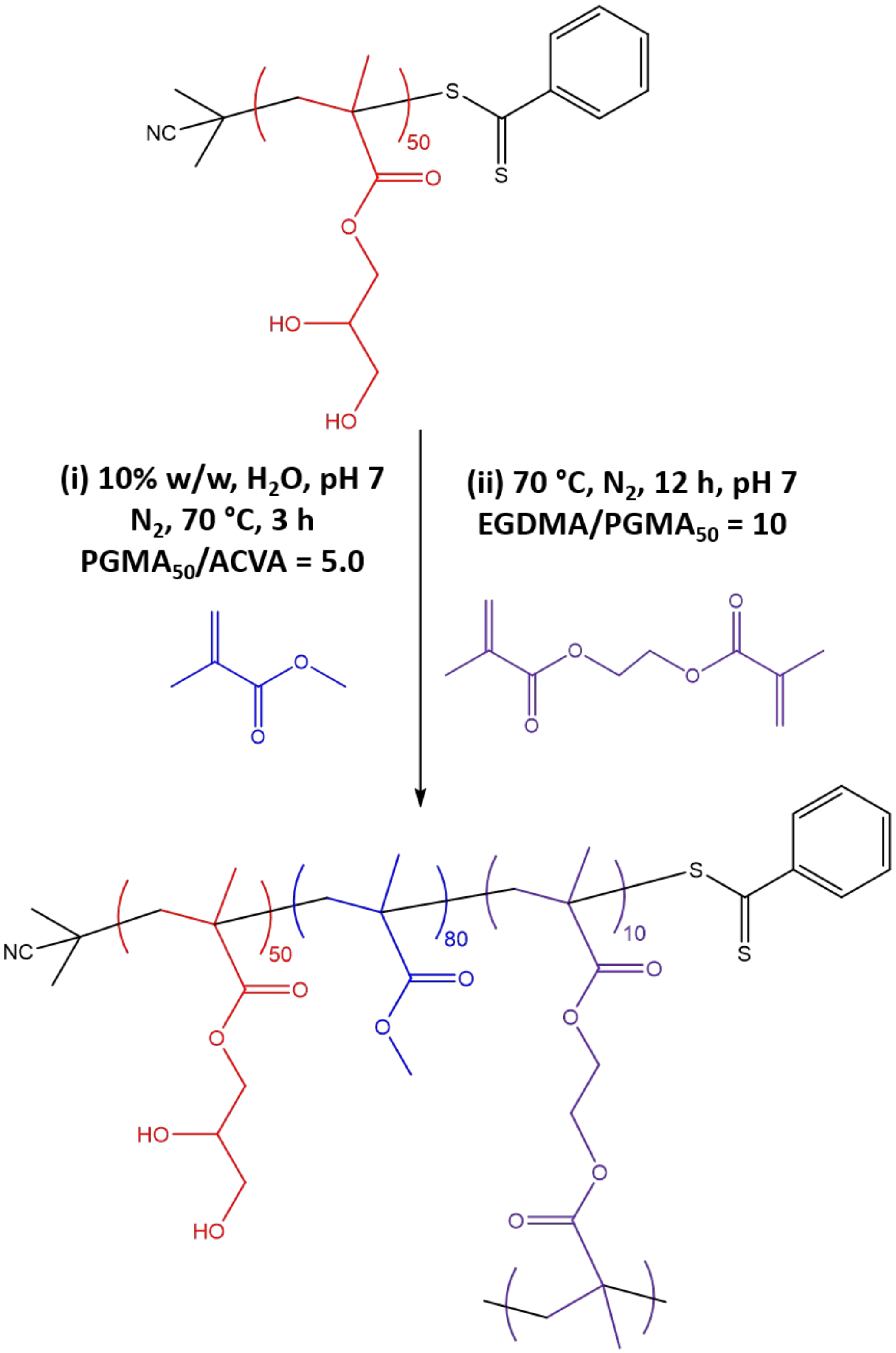

Scheme S1. Schematic synthesis of core-crosslinked $PGMA_{50}$-$PMMA_{80}$-$PEGDMA_{10}$ triblock copolymer nanoparticles via sequential RAFT aqueous emulsion polymerization of (i) MMA and (ii) EGDMA at 70 °C.

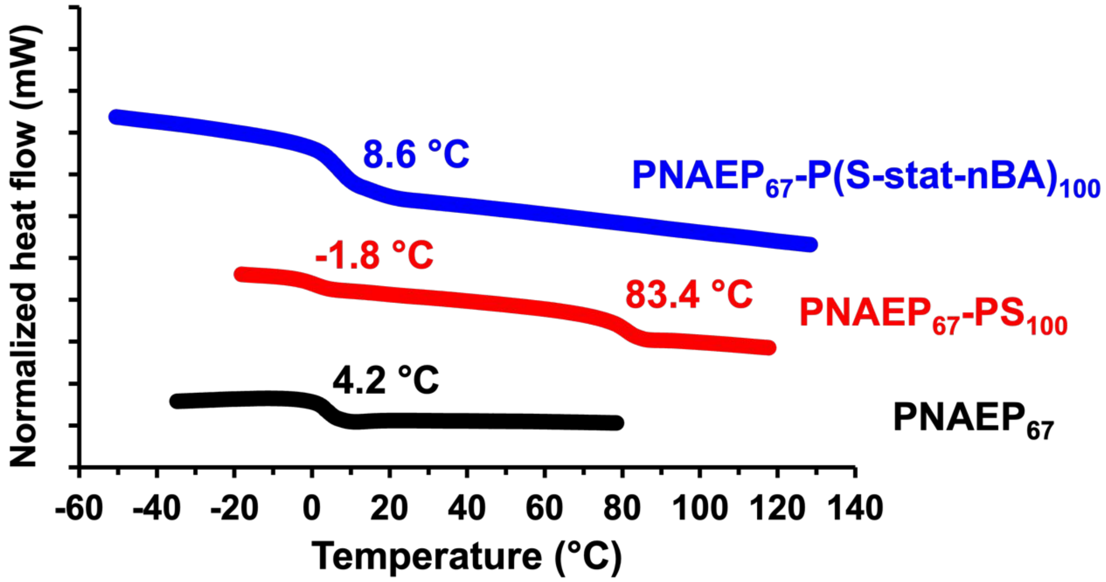

Figure S3. Differential scanning calorimetry (DSC) curves recorded for dried $PNAEP_{67}$-PS_{100} nanoparticles, dried $PNAEP_{67}$-P(S-*stat*-nBA)$_{100}$ nanoparticles (nBA = 55% by mass) and the $PNAEP_{67}$ precursor.

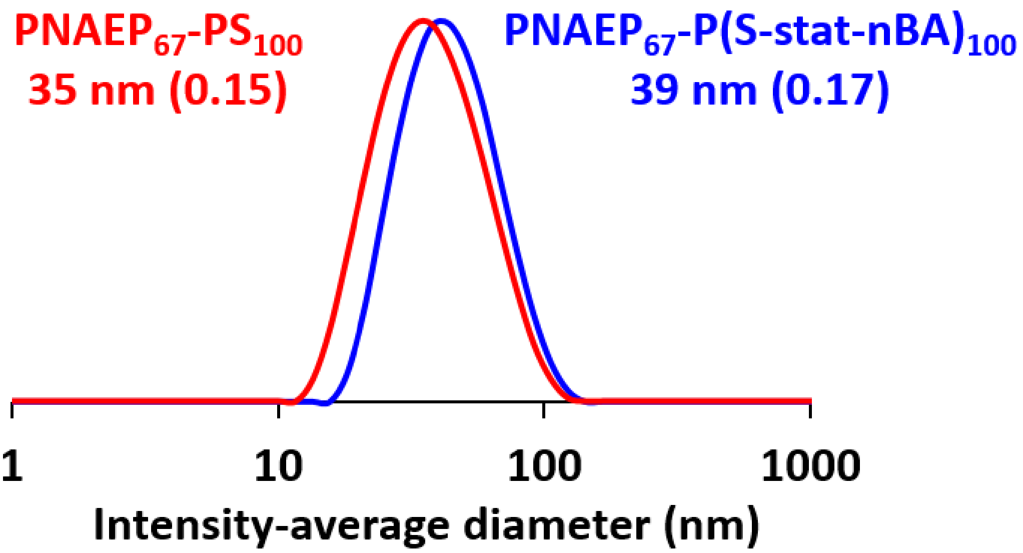

Figure S4. DLS particle size distributions recorded for 0.1% w/w aqueous dispersions of $PNAEP_{67}$-PS_{100} and $PNAEP_{67}$-P(S-*stat*-nBA)$_{100}$ nanoparticles.

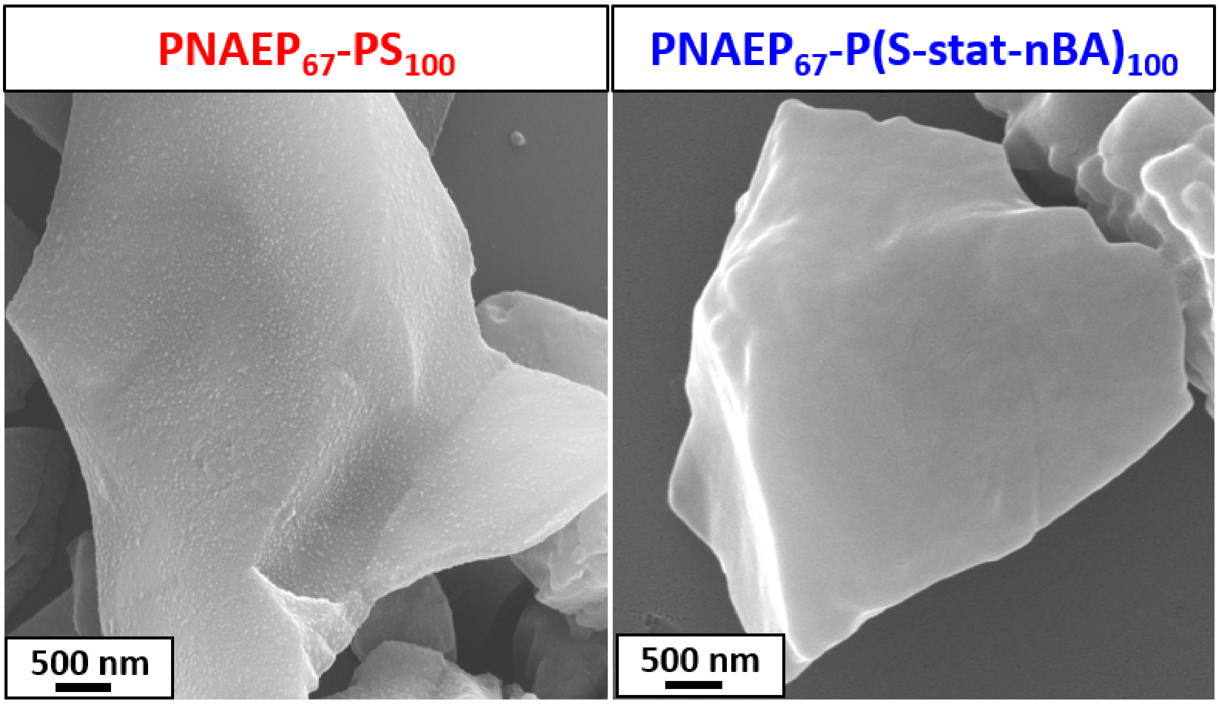

Figure S5. SEM images recorded for azoxystrobin microparticles prepared by milling in the presence of either $PNAEP_{67}$-PS_{100} or $PNAEP_{67}$-P(S-*stat*-nBA)$_{100}$ nanoparticles after removal of excess nanoparticles by centrifugation.

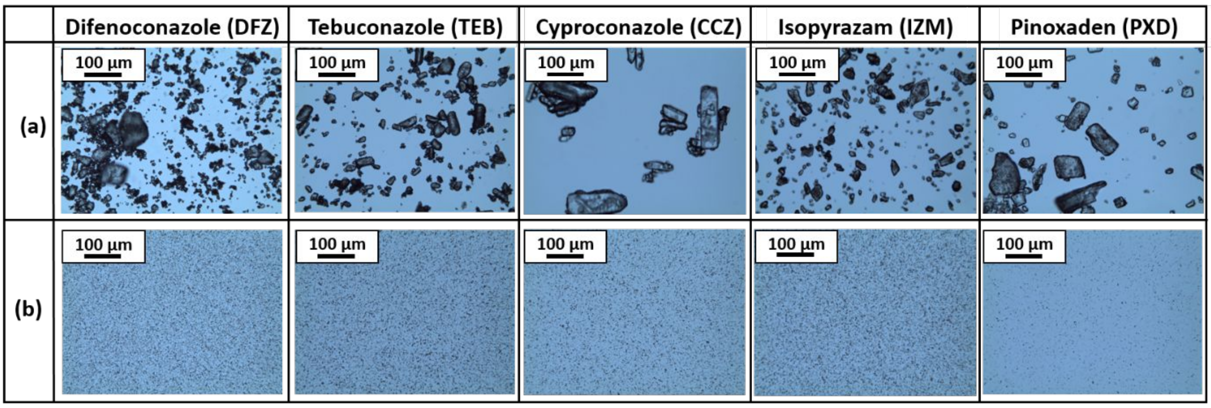

Figure S6. Optical microscopy images recorded for the following five active ingredients (a) before ball milling and (b) after ball milling using the tube drive: difenoconazole, tebuconazole, cyproconazole, isopyrazam and pinoxaden. In these experiments, ball milling of each active ingredient was performed in the presence of $PGMA_{50}$-$PMMA_{80}$ nanoparticles (z-average diameter = 29 nm).
